# Cultural narratives, social norms, and psychological stigma: a study of mental health help-seeking behavior in Peshawar, Pakistan

**DOI:** 10.3389/fpsyt.2025.1560460

**Published:** 2025-08-20

**Authors:** Umar Daraz, Štefan Bojnec, Younas Khan, Zakir Hussain

**Affiliations:** ^1^ Assistant Professor, Department of Sociology, University of Malakand, Chakdara, Pakistan; ^2^ Faculty of Management, University of Primorska, Koper-Capodistria, Slovenia; ^3^ Faculty of Economics and Management, Institute of Economic Policy and Finance, Slovak University of Agriculture, Nitra, Slovakia; ^4^ Department of Economics, Faculty of Economics and Management, Czech University of Life Sciences Prague, Prague, Czechia; ^5^ Department of Management Science and Engineering, School of Business, East China University of Science and Technology, Shanghai, China; ^6^ Lecturer, Department of Social Work, University of Malakand, Chakdara, Pakistan

**Keywords:** cultural narratives, social norms, mental health stigma, psychiatric help, structural equation modeling (SEM)

## Abstract

**Introduction:**

Mental health stigma remains a major barrier to accessing psychiatric care worldwide, with pronounced effects in culturally traditional societies such as Peshawar, Pakistan. In the Pashtun cultural context, the code of Pashtunwali—an honor-based system—shapes social attitudes and behaviors, potentially influencing mental health help-seeking patterns. This study examines how cultural narratives, social norms, and stigma interact to affect help-seeking behavior in this sociocultural setting.

**Methods:**

A cross-sectional survey was conducted among a stratified random sample of 400 adults aged 19 years and above in Peshawar. Data were collected using culturally validated instruments, including the Mental Illness Stigma Scale (MISS) and a Social Norms Scale. Bivariate analyses employed simple linear regression and binary logistic regression to examine individual relationships between variables. Multivariate analyses, including multiple linear regression and Structural Equation Modeling (SEM), were used to assess combined effects and mediation pathways.

**Results:**

Cultural narratives had a positive impact on help-seeking behavior, explaining 42% of its variance. Stigma showed a significant negative association, decreasing help-seeking likelihood by 26% for each unit increase. Social norms demonstrated a positive association with help-seeking behavior and indirectly reduced stigma. Collectively, these variables accounted for 68% of the variance in help-seeking likelihood.

**Discussion:**

The findings highlight the pivotal role of culturally resonant narratives and supportive social norms rooted in Pashtunwali in improving mental health service utilization. Addressing stigma while reinforcing positive cultural frameworks can substantially enhance help-seeking behavior in Peshawar and similar sociocultural contexts.

## 1 Introduction

Mental health is a vital component of overall well-being, yet stigma surrounding mental illness continues to pose a significant barrier to psychiatric care across the globe. In many parts of the world, cultural narratives and social norms frame mental illness not as a medical condition but as a moral failing, spiritual weakness, or personal defect (1, 2). These views discourage help-seeking behavior, resulting in delayed diagnosis, untreated conditions, and deteriorating mental health outcomes (3, 4). According to the World Health Organization (WHO), nearly 75% of individuals with mental health disorders in low- and middle-income countries do not receive the care they need, largely due to stigma and culturally driven misconceptions (5, 6). While global awareness campaigns and mental health reforms have made some headway, their effectiveness varies widely depending on local socio-cultural contexts.

In Pakistan, mental health stigma is compounded by religious beliefs and cultural narratives that often portray mental illness as divine punishment, black magic, or familial shame. Approximately 15–20% of the Pakistani population experiences mental health disorders, yet a vast majority avoid seeking psychiatric help (7, 8). Islamic teachings, though encouraging compassion and healing, are often misinterpreted in local discourse, leading to beliefs that mental illness results from weak faith or possession by jinns (9, 10). Similar patterns of stigma have been documented in other Muslim-majority countries, such as Egypt, Saudi Arabia, and Indonesia, where mental illness is frequently attributed to supernatural causes, and psychiatric help is viewed as secondary to religious healing (11–13).

Despite progress in metropolitan areas like Karachi and Lahore, conservative provinces such as Khyber Pakhtunkhwa remain significantly underserved and under-researched. The province faces an acute shortage of psychiatrists, with fewer than 50 mental health professionals serving a population of over 35 million (14). Peshawar, the capital of Khyber Pakhtunkhwa, exemplifies this mental health gap. While several non-governmental organizations (NGOs) and the Lady Reading Hospital have launched limited psychiatric services, Pakistan lacks an integrated, community-based mental health service model, especially in conservative areas (15, 16). The biomedical model dominates service delivery, with little attention to social, cultural, and religious dimensions, which may inadvertently reinforce stigmatization due to poor cultural alignment.

Peshawar, as a cultural hub of the Pashtun ethnic group, is governed by *Pashtunwali*, a traditional code of conduct that includes values such as *nang* (honor), *purdah* (privacy/modesty), *badal* (revenge), and *melmastia* (hospitality). These values, while central to communal life, conflict with open disclosure of mental illness, which may be perceived as dishonorable or shameful (17). *Pashtunwali* promotes resilience and stoicism, discouraging emotional expression or vulnerability. Thus, mental illness is often hidden within families to preserve honor, leading to social isolation and avoidance of formal psychiatric help (18).

The conservative culture in Peshawar further influences mental health stigma through entrenched gender norms, family hierarchies, and spiritual interpretations. For example, women are disproportionately affected due to restrictions on mobility and lack of autonomy in health decisions (19). Educational institutions rarely include mental health in curricula, and religious leaders often dominate local discourse, sometimes framing mental distress as a test from God or a failure of faith (20). Such narratives promote spiritual healing as the first line of treatment, often through peers (saints), *hakeems* (herbalists), and religious shrines (21, 22).

The motivation for this study stems from the urgent need to understand and address mental health stigma in culturally conservative and religiously sensitive contexts like Peshawar. While existing research highlights general patterns across Pakistan, there is a dearth of localized empirical studies that systematically examine the intersection of cultural narratives, social norms, and mental health stigma, particularly within the Pashtun framework. The consequences of untreated mental health conditions—ranging from suicide to intergenerational trauma—underscore the need for context-sensitive mental health policies that acknowledge both structural and socio-cultural barriers.

**Figure 1:**
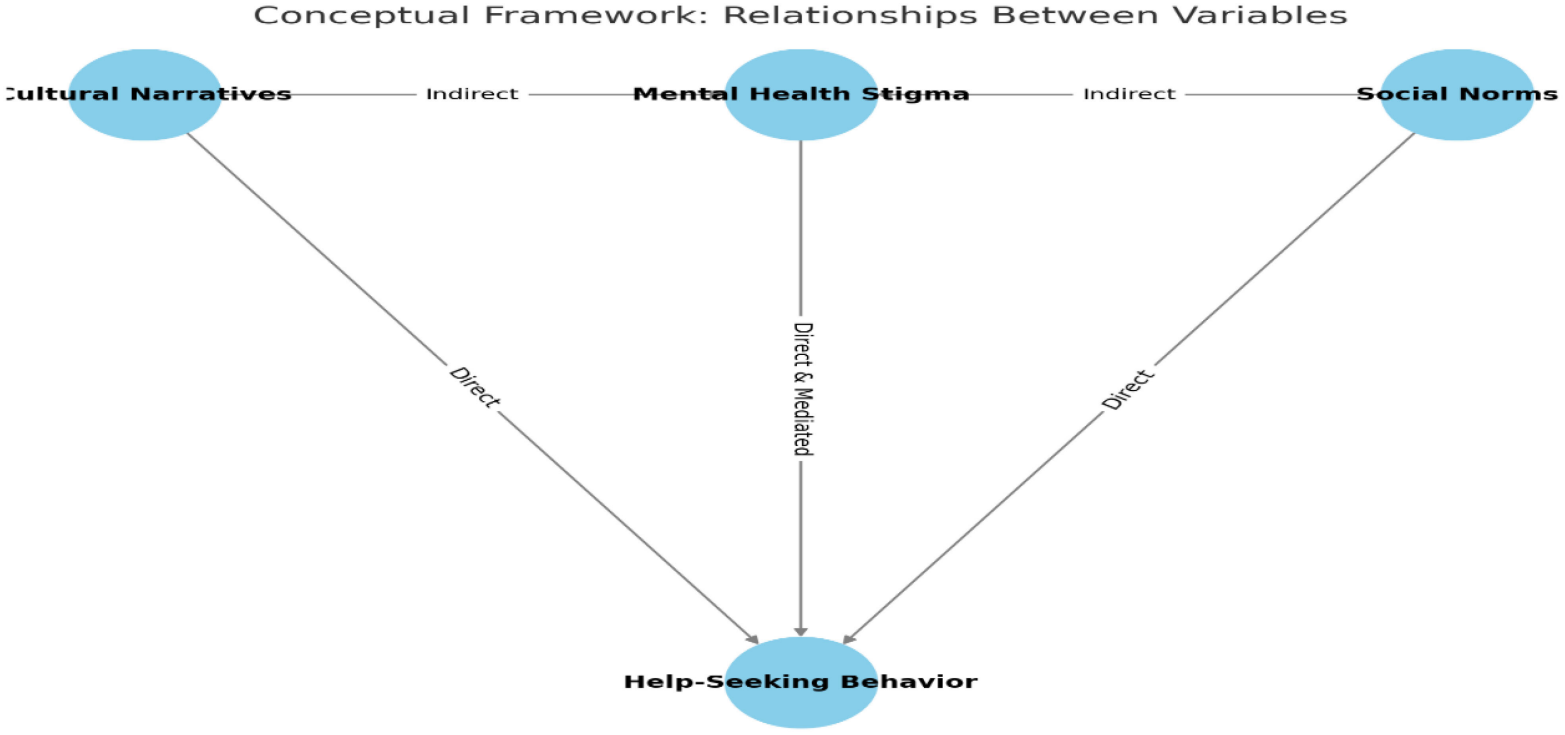
Conceptual framework.

To explore these dynamics, the study draws on a conceptual framework that identifies cultural narratives, social norms, and mental health stigma as key predictors of psychiatric help-seeking behavior (**Figure 1**). Cultural narratives function as belief systems that define mental illness, while social norms influence what behaviors are socially acceptable within communities. Stigma is analyzed as both a product of these narratives and a mediator in the relationship between norms and help-seeking behavior. These relationships are empirically assessed using quantitative methods, including simple regression, logistic regression, multivariate regression, and Structural Equation Modeling (SEM), to measure both direct and indirect effects.

Substantial evidence supports this framework. Yang et al. (23) show that in collectivist cultures, concerns about family honor suppress mental health disclosure. Gregg (24) and Rathod et al. (25) argue that stigma in conservative societies is perpetuated by religious dogmas and social silence. In Peshawar, Ali (17) found that families often conceal mental illness to avoid reputational damage. Choudhry et al. (21) and Maloušková and Fafejta (22) observed widespread preference for spiritual remedies over psychiatric services. Despite these insights, few studies have examined these variables within a structured framework in conservative cities like Peshawar—highlighting a significant research gap this study aims to fill.

The following hypotheses guide this investigation:

- H_1_ (Cultural Narratives): Cultural narratives significantly influence the likelihood of seeking psychiatric help.
- H_2_ (Mental Health Stigma): Mental health stigma significantly reduces the likelihood of seeking psychiatric help.
- H_3_ (Social Norms): Social norms significantly influence the likelihood of seeking psychiatric help.
- H_4_ (Combined Effects): The combined effects of cultural narratives, mental health stigma, and social norms significantly predict seeking psychiatric help.

## 2 Materials and methods

### 2.1 Research design

This study employed a cross-sectional research design to investigate the relationships among cultural narratives, social norms, mental health stigma, and help-seeking behaviors within the socio-cultural context of Peshawar, Khyber Pakhtunkhwa. This design was suitable for capturing a snapshot of how these variables interact at a single point in time (20). The research aimed to examine not only how cultural narratives and social norms contribute to mental health stigma but also how stigma, in turn, mediates the relationship between these socio-cultural constructs and individuals’ willingness to seek professional psychiatric help. By positioning mental health stigma as both an outcome of cultural and social influences and a key predictor of help-seeking behavior, the design facilitated the identification of measurable direct and indirect associations using quantitative analysis. This approach aligns with the broader objective of understanding how context-specific cultural and normative beliefs shape mental health behaviors in conservative settings like Peshawar.

### 2.2 Study setting

The study was conducted in urban and peri-urban areas of Peshawar, the provincial capital of Khyber Pakhtunkhwa, Pakistan. Peshawar’s strong cultural adherence to Pashtunwali norms—emphasizing honor, family reputation, and communal values—made it a compelling setting to explore mental health stigma. This setting allowed for a culturally grounded examination of how traditional beliefs influence mental health perceptions and behaviors.

### 2.3 Study population

The study population consisted of adults aged 19 years and above residing in Peshawar, Khyber Pakhtunkhwa. This age threshold was chosen because individuals at this stage are generally recognized as legally and socially autonomous in Pakistan, enabling them to make informed decisions about personal and healthcare matters, including mental health. Additionally, individuals in this age group are more likely to be exposed to academic, occupational, and familial pressures—factors that can intensify mental health challenges and bring stigma into sharper focus.

The target population was drawn from within this broader adult population and included three primary categories: (1) individuals who had personally experienced mental health issues, (2) individuals who had accessed or attempted to access mental health services, and (3) caregivers or close family members of individuals with mental health conditions. These groups were selected based on their potential to provide first-hand insights into the lived experience of mental health challenges and the social and cultural dynamics influencing help-seeking behavior.

Participants were also selected based on their experiential relevance to the topic—that is, their personal or relational connection to mental health experiences. This inclusive approach allowed the study to capture a diverse range of perspectives on how cultural narratives, social norms, and stigma intersect to influence decisions about seeking psychiatric care. In doing so, the research aimed to reflect the collective understanding of mental health perceptions across different roles within the community, offering a more nuanced and comprehensive exploration of the issue.

### 2.4 Sampling procedures and sample size

A stratified random sampling technique was employed to ensure balanced representation across key demographic subgroups within Peshawar. The stratification process was guided by three primary variables: gender (male/female), education level (no formal education, primary/secondary, tertiary), and socio-economic status (low, middle, high income). These strata were chosen because previous research indicates that perceptions of mental health and help-seeking behavior can vary significantly across these demographic lines (26).

To implement this technique:

1. Strata Formation: The total adult population of Peshawar was first divided into mutually exclusive strata based on the three variables (gender, education, and income). For example, male respondents with tertiary education from low-income backgrounds formed one stratum, while female respondents with no formal education from middle-income households formed another.
2. Proportional Allocation: The number of respondents selected from each stratum was determined proportionally to the size of that subgroup in the population. This ensured that larger subgroups (e.g., middle-income individuals or those with secondary education) were appropriately represented without over- or under-sampling smaller groups.
3. Random Selection Within Strata: Once strata were established and proportions set, simple random sampling was used within each stratum to select individuals. This helped minimize selection bias and ensured that each eligible person within a stratum had an equal chance of being included in the sample.

According to the 2017 Pakistan Population Census, the estimated adult population (aged 19 and above) in Peshawar was approximately 1.2 million. Given the size and variability of this population—and accounting for ongoing fluctuations due to migration and population growth—the population was treated as effectively infinite for sampling purposes. Using Cochran’s formula for large populations:


$$n=z^{2}\cdot p\cdot (1-p)/e^{2}$$

Where:

Z= 1.96 (for a 95% confidence level).

p = 0.5 (assumed proportion of the population with a characteristic of interest, as this gives the maximum sample size).

e = 0.05 (margin of error).


$$n=\;{\left(1.96\right)}^{2}.\;0.5.\;\left(1-0.5\right)/{\left(0.05\right)}^{2}=\;384.16$$

Since the population is finite, the adjusted sample size is calculated using:


$$n_{adjusted}=n/1+n/N$$

Where:

n = 384.16 (initial sample size).

N = 1,200,000 (target population).


$$n_{adjusted}=384.16/1\;+\;384.16/1,200,000\;=\;384$$

To strengthen the robustness of the analysis, the sample size was increased from 384 to 400 based on the following statistical and methodological considerations:

- Anticipated Non-Response: A modest buffer was added to account for potential non-responses, incomplete questionnaires, or data quality issues, ensuring that the final usable dataset would remain statistically valid.
- Increased Statistical Power: A slightly larger sample enhances the study’s power to detect smaller effect sizes in multivariate and Structural Equation Modeling (SEM) analyses, where model complexity and multiple variables require a larger sample for stable estimates.
- Stratified Sampling Precision: Increasing the sample size allowed for better proportional representation within small subgroups (e.g., females with tertiary education in high-income brackets), reducing standard errors and enhancing subgroup comparisons.

Thus, the final sample size of 400 was determined to ensure adequate representation, improve data reliability, and support more nuanced and statistically sound inferences about mental health stigma and help-seeking behavior across diverse segments of Peshawar’s population.

### 2.5 Tool of data collection

Data were collected using a structured questionnaire comprising four main sections: (1) Demographics, (2) Mental Health Stigma, (3) Social Norms, and (4) Cultural Narratives. The questionnaire was carefully designed to standardize responses and ensure content validity and reliability across a large, diverse sample.

### 2.6 Language and translation process

To ensure comprehension, cultural sensitivity, and inclusivity, the questionnaire was initially developed in English and then translated into Urdu and Pashto, the most widely spoken languages in Peshawar.

The translation process followed a rigorous forward–backward translation method:

1. Forward Translation: The original English questionnaire was translated into Urdu and Pashto by two independent bilingual experts with experience in public health and social research.
2. Expert Review: A panel of local mental health professionals, cultural experts, and linguists reviewed the translated versions to assess cultural appropriateness and conceptual equivalence. Special attention was paid to terms related to mental illness, stigma, and cultural beliefs to avoid misinterpretation or offense.
3. Back-Translation: A separate set of bilingual experts, who had no access to the original English version, retranslated the Urdu and Pashto versions back into English. This allowed for comparison and identification of discrepancies.
4. Pretesting and Refinement: The translated questionnaires were pilot-tested during the first week of data collection with a small subset (n = 20) of participants from the target population. Feedback on clarity, sensitivity, and comprehension was gathered and used to refine problematic items.

### 2.6 Questionnaire sections

- Demographics included items on age, gender, education, income, and employment status.
- Mental Health Stigma was measured using the validated Mental Illness Stigma Scale (MISS) by Lim et al. (27), focusing on stereotyping, discrimination, and social distancing.
- Social Norms were assessed using adapted items from Perkins and Berkowitz (28) Social Norms Scale, which measures perceived normative pressures.
- Cultural Narratives were captured through custom-developed items based on Pashtunwali principles (e.g., honor, shame, spiritual beliefs), developed in collaboration with Pashtun cultural scholars and community leaders.

Additionally, help-seeking behaviors were assessed using relevant items embedded within the stigma and social norms sections, allowing for the evaluation of both direct and mediated effects.

The data collection process spanned four weeks:

- Week 1: Pilot testing and linguistic refinement
- Weeks 2 & 3: Full-scale distribution and weekly reminders
- Week 4: Data cleaning, validation, and exclusion of incomplete responses

### 2.7 Ethical considerations

Ethical approval for the study was obtained from the Institutional Review Board of University of Malakand, Pakistan, with approval number IRB/PSY-2024/044, dated March 5, 2024. All participants gave informed consent prior to participation. Confidentiality and anonymity were strictly maintained by assigning codes to responses, and participants were informed of their right to withdraw at any stage of the research.

### 2.8 Measurement of variables and indexation

To explore the interrelationships among cultural narratives, social norms, mental health stigma, and help-seeking behavior, this study utilized validated, culturally adapted instruments. Variables were operationalized through composite indices to enable robust statistical testing, including regression and SEM. In accordance with the study’s conceptual framework, mental health stigma functioned as a mediating variable, influenced by cultural narratives and social norms, and influencing help-seeking behavior.

### 2.9 Cultural narratives (independent variable)

Cultural narratives were measured using custom-developed survey items that captured dominant beliefs within the Pashtun cultural context—particularly those associated with honor (nang), communal obligations, and gendered expectations. The items were developed through consultation with cultural experts and pilot-tested for clarity and contextual relevance. Responses were collected using a 5-point Likert scale, and aggregated into a composite Cultural Narrative Index, with higher scores indicating stronger adherence to traditional cultural narratives.

### 2.10 Social norms (independent variable)

Social norms were assessed using a culturally adapted version of the Social Norms Scale (29), focusing on perceptions of what is considered acceptable regarding emotional expression and mental health support in the community. The scale included Likert-type items that gauged injunctive norms (what people think others approve of) and descriptive norms (what others typically do). Responses were aggregated into a Social Norms Index.

### 2.11 Mental health stigma (mediating variable)

Mental health stigma was measured using the Mental Illness Stigma Scale (MISS), which captures multiple dimensions of stigma, including stereotyping, discrimination, and social distancing (30). The instrument was pre-tested and linguistically adapted for cultural sensitivity in Peshawar. Responses were aggregated to yield a Stigma Index Score, with higher values indicating greater stigma endorsement. As a mediating variable, stigma was modeled to assess how it links cultural narratives and social norms to help-seeking behavior.

### 2.12 Help-seeking behavior (dependent variable)

Help-seeking behavior was evaluated through items adapted from established scales measuring willingness, intent, and previous engagement with mental health services. The items captured both formal (e.g., psychiatrists, psychologists) and informal (e.g., religious or community leaders) help sources. These were also measured on a 5-point Likert scale and indexed to create a Help-Seeking Behavior Score, reflecting participants’ propensity to seek support.

### 2.13 Indexation and scoring

**Figure 2:**
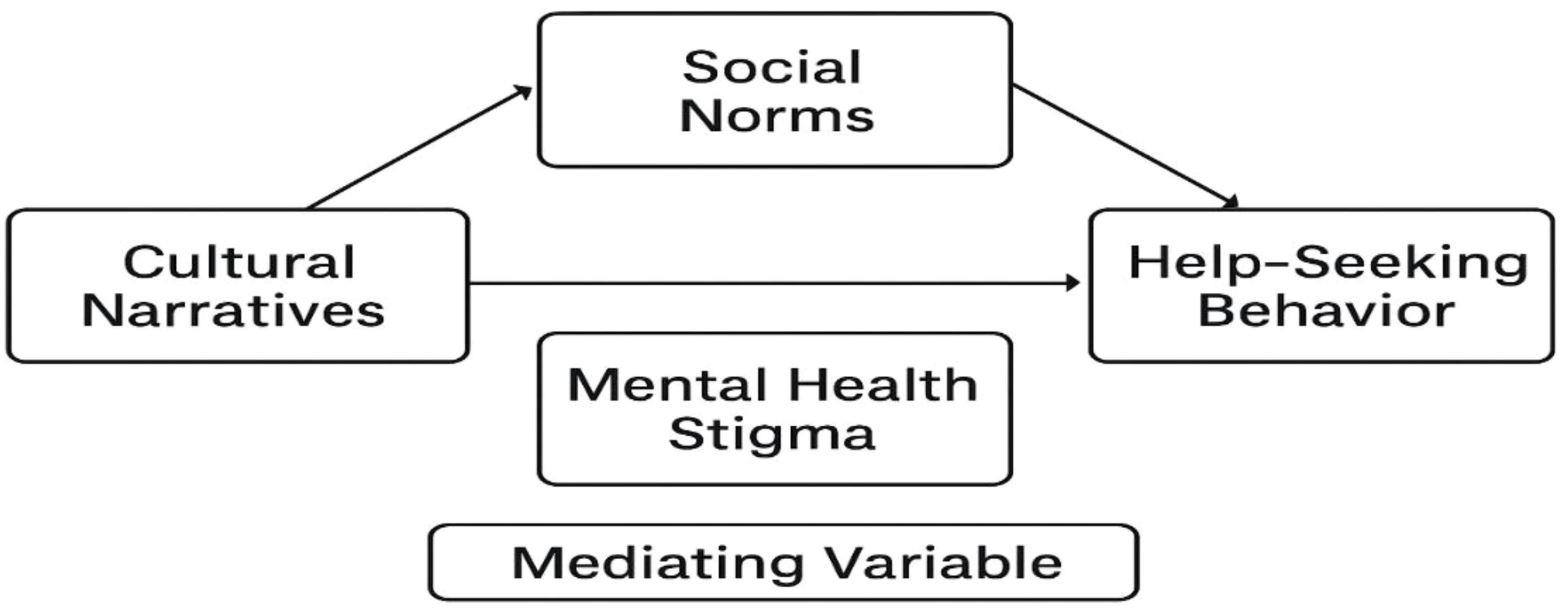
Operational framework.

All variables were indexed by summing or averaging responses across relevant items. Internal consistency of the scales was evaluated using Cronbach’s alpha to ensure reliability. These composite scores enabled quantitative analyses to assess direct and indirect relationships among the constructs. The indexation allowed for meaningful interpretation of cultural and social influences on stigma, and the subsequent impact of stigma on mental health help-seeking behavior (**Figure 2**).

### 2.14 Reliability and validity of the tool

Reliability of the scales was evaluated using Cronbach’s Alpha, with results indicating satisfactory internal consistency (α > 0.7 for all scales). This confirmed the stability and consistency of the items across the sample.

Validity was established through multiple steps:

• Content validity: Achieved via expert review from professionals in mental health and cultural studies.

• Construct validity: Confirmed through Exploratory Factor Analysis (EFA), demonstrating alignment with the theoretical dimensions of stigma, norms, and narratives.

• Pilot testing: Provided empirical feedback to ensure clarity and cultural appropriateness, leading to necessary adjustments before full deployment.

### 2.15 Data analysis and models of the study

Data analysis was conducted using SPSS version 26, employing both bivariate and multivariate statistical techniques to test the study’s hypotheses. In the bivariate analysis phase, individual relationships between variables were assessed through simple regression, logistic regression, and SEM. These methods provided initial insights into how specific cultural narratives and social norms relate to mental health stigma and help-seeking behaviors. The multivariate analysis explored the combined and interactive effects of multiple independent variables on help-seeking behavior. This was achieved through multiple regression, path analysis, and SEM, which enabled a more comprehensive understanding of both direct and mediated pathways. These advanced techniques allowed for the testing of the conceptual framework developed in the study, confirming the mediating role of stigma between cultural norms and help-seeking behavior.

The models for both bivariate and multivariate analyses are detailed below:

Models for Bivariate Analysis (Hypotheses 1, 2 and 3)

Model 1: Bivariate Regression Analysis: To evaluate the influence of Cultural Narratives on Seeking Psychiatric Help (Hypothesis 1).

Equation: Y=β0+β1X1+∈

  Y: Seeking Psychiatric Help (Dependent Variable)
β0: Constant (Intercept)
β1: Coefficient for Cultural Narratives
X1: Cultural Narratives (Independent Variable)
∈: Error term

Model 2: Logistic Regression Analysis (Bivariate): To determine the likelihood of Seeking Psychiatric Help based on Mental Health Stigma (Hypothesis 2).

Equation: ln(P(Y=1)/1−P(Y=1))=β0+β1X2 P(Y=1): Probability of seeking psychiatric help.
ln: Natural logarithm of the odds ratio.
β0: Constant (Intercept).
β1: Coefficient for Mental Health Stigma.
X2: Mental Health Stigma (Independent Variable).

Model 3: Structural Equation Modeling (SEM - Bivariate): To explore the direct and indirect effects of Social Norms and Mental Health Stigma on Seeking Psychiatric Help (Hypothesis 3).

Path Equations:

1. Direct Path: Y=β1X3

Y: Seeking Psychiatric Help (Dependent Variable)
X3: Social Norms (Independent Variable)
β1: Path coefficient representing the direct effect of X3 on Y.
2. Indirect Path: X4=β2X3, Y=β3X4

  X4: Mental Health Stigma (Mediator Variable)
β2: Path coefficient representing the effect of X3 on X4.
β3: Path coefficient representing the effect of X4 on Y.

Models for Multivariate Analysis (Hypothesis 4)

Model 4: Multivariate Regression Analysis: To assess the combined effects of Cultural Narratives, Mental Health Stigma, and Social Norms on Seeking Psychiatric Help (Hypothesis 4).

Equation: Y=β0+β1X1+β2X2+β3X3+∈

  Y: Seeking Psychiatric Help (Dependent Variable).
β0: Intercept (constant term in the regression equation).
β1, β2, β3: Regression coefficients for the independent variables.
β1: Effect of Cultural Narratives (X1) on Y
β2: Effect of Mental Health Stigma (X2) on Y.
β3: Effect of Social Norms (X3) on Y.
X1: Cultural Narratives (Independent Variable 1).
X2: Mental Health Stigma (Independent Variable 2).
X3: Social Norms (Independent Variable 3).
∈: Error term or residual, accounting for variability in Y not explained by the independent variables.

Model 5: Path Analysis and SEM (Multivariate): To examine the direct and indirect effects of Cultural Narratives, Mental Health Stigma, and Social Norms on Seeking Psychiatric Help (Hypothesis 4).

Direct Effect Equations:

1. Cultural Narratives → Seeking Psychiatric Help: Y=β1X1
2. Mental Health Stigma → Seeking Psychiatric Help: Y=β2X2
3. Social Norms → Seeking Psychiatric Help: Y=β3X3

Indirect Effect Equations (Mediation):

1. Cultural Narratives → Mental Health Stigma → Seeking Psychiatric Help: X2=β4X1, Y=β5X2 2. Social Norms → Mental Health Stigma → Seeking Psychiatric Help: X2=β6X3,Y=β7X2 Y: Seeking Psychiatric Help (Dependent Variable).
X1: Cultural Narratives (Independent Variable).
X2: Mental Health Stigma (Mediator).
X3: Social Norms (Independent Variable).
β1, β2, β3: Standardized coefficients for direct effects of Cultural Narratives, Mental Health Stigma, and Social Norms on Seeking Psychiatric Help.
β4, β5, β6, β7: Standardized coefficients for indirect paths (mediation effects).

## 3 Results

### 3.1 Socio-economic and demographic features of respondents

The socio-economic and demographic variables selected for this study are integral to understanding how cultural narratives and social norms shape mental health stigma and influence help-seeking behaviors within the unique cultural context of Peshawar, Khyber Pakhtunkhwa. These characteristics provide critical insight into the ways individuals internalize or resist culturally embedded beliefs about mental health (**Table 1**).

**Table 1:** Socio-economic and demographic characteristics of the respondent.

Variable	Categories	Sample size	Total
Age	• 19–29 Years • 30–44 Years • 45 and Above	122 157 121	400
Gender	• Male • Female	200 200	400
Education	• No formal education • Secondary education • Higher Education	76 118 226	400
Income PKR	• Less than 30,000 • 30,000–60,000 • Above 60, 000	95 155 150	400
Employment Status	• Employed • Unemployed	263 133	400

Age plays a pivotal role, as it intersects with generational exposure to changing societal norms. Younger respondents, often more exposed to global media and education, may adopt progressive attitudes that challenge traditional stigmatizing views. In contrast, older individuals are more likely to adhere to culturally entrenched norms rooted in Pashtunwali, potentially reinforcing stigma and discouraging formal help-seeking.

Gender, a deeply embedded construct in Pashtun culture, significantly shapes the perception of mental health and responses to psychological distress. Women may experience dual stigma—first for the mental health condition itself, and second for transgressing gender norms that expect emotional resilience and silence. Men, conversely, may avoid help-seeking due to societal expectations that equate masculinity with emotional suppression and self-reliance, reinforcing stigma through cultural narratives that discourage vulnerability.

Education level serves as a strong predictor of mental health literacy. Individuals with higher education are typically more aware of scientific understandings of mental illness and alternative cultural narratives, thereby reducing the internalization of stigma and increasing the likelihood of help-seeking. Education facilitates critical engagement with social norms and fosters openness to diverse mental health discourses.

Income level directly influences access to mental health services and information. Those with higher income not only possess better access to private mental health care but may also experience less stigma due to the empowerment that economic security brings. Conversely, low-income individuals may be more vulnerable to cultural narratives that associate mental illness with weakness or divine punishment, especially in contexts where economic hardship is already stigmatized.

Employment status reflects both economic independence and societal role fulfillment, which are important within the cultural framework of Pashtun society. Unemployed individuals may face amplified stigma, as mental health issues are often perceived as undermining one’s capacity to fulfill social and familial responsibilities. This intersection reinforces negative cultural narratives and further discourages help-seeking behaviors.

### 3.2 Bivariate analysis

#### 3.2.1 Testing hypothesis H_1_: cultural narratives and help-seeking behavior

The results of the bivariate regression analysis presented in **Table 2** offer significant insights into the relationship between cultural narratives and help-seeking behavior within the sociocultural context of Peshawar, Pakistan. The regression coefficient for cultural narratives (B = 0.60) indicates that for every one-unit increase in the cultural narratives score, the help-seeking behavior score increases by 0.60 units. This suggests a positive and statistically significant influence of cultural narratives on the likelihood of seeking psychiatric help among individuals in Peshawar.

**Table 2:** Regression analysis (bivariate).

Regression coefficients						
Predictor	B (Unstandardized)	SE (Standard error)	Beta (Standardized)	T-value	P-value	95% Confidence interval
Constant	1.80	0.50	–	3.60	0.000	[0.81, 2.79]
Cultural Narratives Score	0.60	0.08	0.65	7.50	0.000	[0.44, 0.76]
ANOVA						
Model	Sum of Squares	df	Mean Square	F		p-value
Regression	118.80	1	118.80	56.25		0.000
Residual	163.20	398	0.41	–		–
Total	282.00	399	–	–		–
Model summary						
Model	R	R²	Adjusted R²	Standard Error (SE)		
1	0.65	0.42	0.41	0.75		

The standardized coefficient (Beta = 0.65) highlights the strong effect size, indicating that cultural narratives account for a substantial proportion of the variability in help-seeking behavior. These findings are further supported by a t-value of 7.50 and a p-value of 0.000, which are well below the conventional significance threshold of 0.05. This confirms that the observed relationship is statistically significant and unlikely due to random chance.

The ANOVA results reinforce the overall model validity, with an F-value of 56.25 and a p-value of 0.000, establishing that the inclusion of cultural narratives in the model significantly improves the prediction of help-seeking behavior. The coefficient of determination (R² = 0.42) reveals that 42% of the variance in help-seeking behavior is explained by cultural narratives, indicating a moderate to strong explanatory power. Additionally, the adjusted R² = 0.41 accounts for the model’s complexity, confirming the stability and reliability of the regression results.

Moreover, the 95% confidence interval [0.44, 0.76] for the unstandardized regression coefficient provides further evidence that the true population parameter lies within this positive range, substantiating the robustness of the observed relationship.

These findings are consistent with the study’s theoretical framework, which asserts that cultural narratives shape individuals’ mental health beliefs and practices, particularly in collectivist and traditional societies like that of Peshawar. The results indicate that cultural narratives—such as community stories, religious teachings, and local idioms of distress—play a critical role in shaping how mental health is perceived, thus influencing whether individuals seek professional psychiatric help.

In alignment with the study’s overarching aim and the proposed hypothesis one (H_1_), the regression analysis provides strong support for the hypothesis (H_1)_ that cultural narratives significantly influence the likelihood of seeking psychiatric help. These results not only validate the research hypothesis but also underscore the importance of culturally grounded interventions in mental health policy and practice. Recognizing and incorporating culturally relevant narratives can be pivotal in reducing stigma, enhancing mental health literacy, and promoting service utilization in culturally rich and diverse contexts like Peshawar. The study thus contributes to a nuanced understanding of how social norms, cultural values, and stigma interrelate to shape mental health behavior in this region.

#### 3.2.2 Testing hypothesis H_2_: mental health stigma and help-seeking behavior

The logistic regression analysis results presented in **Table 3** offer robust evidence regarding the impact of mental health stigma on the likelihood of seeking psychiatric help among individuals in Peshawar, Pakistan. The regression coefficient for mental health stigma is B = -0.30, indicating a negative relationship. This suggests that with every one-unit increase in mental health stigma, the log-odds of seeking psychiatric help decrease by 0.30 units.

**Table 3:** Logistic regression (bivariate).

Logistic regression coefficients						
Predictor	B (Unstandardized)	SE (Standard error)	Wald	Exp (B) (Odds ratio)	95% CI for exp (B)	P-value
Constant	1.80	0.50	12.96	6.05	2.23 – 16.42	0.000
Mental Health Stigma Score	-0.30	0.05	36.00	0.74	0.65 – 0.83	0.000
Omnibus tests of model coefficients						
Chi-Square		Df (Degree of Freedom)		p-value		
58.25		1		0.000		
Model summary						
Statistic				Value		
-2 Log Likelihood				480.56		
Cox & Snell R²				0.21		
Nagelkerke R²				0.28		

Further, the odds ratio (Exp(B) = 0.74) implies that each unit increase in stigma is associated with a 26% decrease in the odds of an individual seeking psychiatric assistance (i.e., 1 - 0.74 = 0.26). This finding clearly supports the notion that higher stigma is a significant barrier to mental health service utilization in this cultural context.

The Wald statistic = 36.00 with a p-value = 0.000 confirms that the coefficient for mental health stigma is highly statistically significant, reinforcing the conclusion that this relationship is not due to random variation. Additionally, the 95% confidence interval for Exp(B) ranges from 0.65 to 0.83, excluding the value of 1, which further confirms the consistency and reliability of the negative effect of stigma on help-seeking behavior.

Support for the model’s adequacy is further evident in the Omnibus Test of Model Coefficients, which yields a Chi-square value of 58.25 with a p-value = 0.000, demonstrating that the inclusion of mental health stigma significantly improves the model’s explanatory power.

The logistic regression model summary provides additional support, indicating that Cox & Snell R² = 0.21 and Nagelkerke R² = 0.28. This means that between 21% and 28% of the variance in help-seeking behavior is explained by mental health stigma alone—an important and meaningful contribution in the field of mental health behavioral research. Moreover, the -2 Log Likelihood value of 480.56 further indicates an acceptable model fit, reinforcing its predictive strength.

These results are highly consistent with the conceptual framework of the study, which positions mental health stigma as a key socio-psychological barrier that prevents individuals from seeking psychiatric assistance. In the context of Peshawar’s sociocultural environment, where traditional beliefs and fear of social judgment often dominate mental health discourse, this finding reflects the real-world influence of stigma on mental health decision-making.

The findings provide strong empirical support for the hypothesis two (H_2_), which posits that mental health stigma significantly reduces the likelihood of seeking psychiatric help. Moreover, these results reinforce the importance of addressing mental health stigma not as an isolated factor but as interconnected with broader cultural narratives and social norms. The findings underscore how stigma operates both directly and indirectly, shaping public attitudes and individual behaviors regarding mental health care utilization in Peshawar.

#### 3.2.3 Testing hypothesis H_3_: social norms and help-seeking behavior

The results in **Table 4** derived from the SEM analysis provide robust support for Hypothesis 3 (H_3_), which posits that social norms significantly influence the likelihood of seeking psychiatric help. This hypothesis aligns directly with the broader aim of the study—to investigate how cultural narratives and social norms in Peshawar, Pakistan, impact mental health help-seeking behavior through stigma-related mechanisms. The SEM output reveals that social norms exert a significant positive influence on help-seeking behavior, as evidenced by a standardized path coefficient (β) of 0.42, a critical ratio (CR) of 5.25, and a p-value of 0.000. This statistically significant effect indicates that more supportive and accepting social norms are associated with a greater likelihood of individuals in Peshawar seeking psychiatric assistance. These findings substantiate the hypothesis and reflect the powerful role that community-held beliefs and social expectations play in shaping behavioral health outcomes in this cultural context.

**Table 4:** Structural equation modeling (bivariate).

Path coefficients with direct effect				
Path	Standardized coefficient (β)	Standard error (SE)	Critical ratio (CR)	p-value
Social Norms → Seeking Psychiatric Help	0.42	0.08	5.25	0.000
Social Norms → Mental Health Stigma	-0.37	0.07	-5.29	0.000
Mental Health Stigma → Seeking Psychiatric Help	-0.45	0.09	-5.00	0.000

Path coefficients with indirect effects				
Mediation path			Indirect effect (β)	P-value
Social Norms → Mental Health Stigma → Seeking Psychiatric Help			0.17	0.001

Model fit indices				
Fit index			Value	Threshold for good fit
Chi-Square (χ²)			45.21	p > 0.05
Degrees of Freedom (df)			28	N/A
χ²/df Ratio			1.61	< 3.0
Comparative Fit Index (CFI)			0.96	> 0.95
Tucker-Lewis Index (TLI)			0.94	> 0.90
Root Mean Square Error of Approximation (RMSEA)			0.045	< 0.06

Further analysis shows that social norms are significantly and negatively associated with mental health stigma, with a standardized coefficient (β) of -0.37, CR of -5.29, and p-value of 0.000. This finding underscores that when cultural narratives and social norms are supportive and inclusive, they contribute to the reduction of stigma surrounding mental illness in Peshawar. The negative association is critical because stigma has long been identified as a primary barrier to mental health service utilization in conservative and collectivist societies. In alignment with the conceptual model, mental health stigma significantly and negatively impacts help-seeking behavior, with a standardized path coefficient (β) of -0.45, a CR of -5.00, and a p-value of 0.000. This suggests that individuals who internalize societal stigma are significantly less likely to pursue psychiatric help. Within the context of Peshawar’s cultural narratives, where mental illness may often be misunderstood or marginalized, this finding highlights the ongoing challenge of stigma reduction.

The indirect effect of social norms on help-seeking behavior—mediated through mental health stigma—is also statistically significant, with a standardized coefficient (β) of 0.17 and a p-value of 0.001. This indirect pathway confirms that social norms influence help-seeking behavior not only directly but also indirectly by mitigating stigma. The presence of this mediation effect supports the study’s framework that positions stigma as a central mechanism through which cultural and societal expectations shape individual health choices in Peshawar. The overall SEM model demonstrates excellent fit with the observed data. The chi-square statistic (χ²) of 45.21 with 28 degrees of freedom results in a χ²/df ratio of 1.61, which is well below the acceptable threshold of 3.0. The Comparative Fit Index (CFI) is 0.96, the Tucker-Lewis Index (TLI) is 0.94, and the Root Mean Square Error of Approximation (RMSEA) is 0.045—all indicating a well-fitting model that accurately captures the relationships among the study variables.

Based on the results, Hypothesis three (H_3_) is strongly supported. The findings confirm that in the sociocultural landscape of Peshawar, supportive social norms play a pivotal role in promoting help-seeking behaviors both directly and indirectly by reducing stigma. These outcomes reinforce the study’s theoretical framework and empirical focus, validating that cultural narratives and social expectations can either hinder or facilitate mental health service utilization. In conclusion, the results of Hypothesis 3 are fully aligned with the study’s objectives, the theoretical assumptions, and the SEM analysis presented in the statistical tables. They provide evidence-based direction for mental health advocacy in Peshawar: interventions aimed at reshaping social norms and cultural narratives may be key to reducing stigma and increasing psychiatric service uptake.

### 3.3 Multivariate analysis

#### 3.3.1 Testing hypothesis H_4_: combined effects of cultural narratives, mental health stigma, and social norms on help-seeking behavior

The results in **Table 5** of the multiple regression analysis provide a comprehensive understanding of the combined influence of cultural narratives, mental health stigma, and social norms on the likelihood of seeking psychiatric help in Peshawar, Pakistan. This analysis directly addresses Hypothesis four (H_4_), which proposes that the joint effects of these three socio-cultural factors significantly predict help-seeking behavior. The coefficients derived from the regression model indicate the strength and direction of each variable’s relationship with the dependent variable, while the standardized coefficients (Beta, β) allow for comparison of their relative contributions.

**Table 5:** Multiple regression (multivariate).

Predictor (IV)		Coefficient (B)	Standard error (SE)	Beta (β)		T-value	P-value
Cultural Narratives		0.52	0.08	0.41		6.50	<0.001
Mental Health Stigma		-0.37	0.06	-0.32		-6.17	<0.001
Social Norms		0.46	0.07	0.38		6.57	<0.001
Constant		1.25	0.12	N/A		10.42	<0.001
Model summary							
R²	Adjusted R²		F-statistic		p-value		
0.68	0.67		85.42		p< 0.001		

Cultural narratives exhibit a positive and statistically significant effect on psychiatric help-seeking behavior, with an unstandardized coefficient (B) of 0.52, a standardized Beta (β) of 0.41, and a t-value of 6.50 (p< 0.001). This suggests that individuals who interpret prevailing cultural narratives in a more supportive and understanding manner are more inclined to pursue professional mental health services. Within the sociocultural setting of Peshawar—where traditional narratives can either endorse or inhibit mental health discussions—this result highlights the vital role of culturally embedded meanings and stories in shaping individual attitudes toward psychiatric help.

Mental health stigma, as anticipated, demonstrates a negative and statistically significant association with help-seeking behavior, as reflected by a coefficient (B) of -0.37, a standardized Beta (β) of -0.32, and a t-value of -6.17 (p< 0.001). These findings confirm that higher levels of perceived stigma are linked with a lower likelihood of seeking psychiatric assistance. This inverse relationship reinforces the critical role of stigma in obstructing access to mental health services and validates the study’s premise that stigma is a powerful psychological and social deterrent, especially in conservative societies like Peshawar, where mental health concerns are often hidden due to fear of social judgment.

Social norms also contribute positively and significantly to help-seeking behavior, with a coefficient (B) of 0.46, a standardized Beta (β) of 0.38, and a t-value of 6.57 (p< 0.001). This indicates that when individuals perceive their surrounding community as supportive of mental health care, they are more likely to seek psychiatric help. In the Peshawar context, where communal values and social expectations heavily influence personal decisions, these findings emphasize the importance of normative beliefs in guiding health-seeking actions. Supportive social norms can counteract stigma and empower individuals to access the care they need.

The constant term in the regression model, with a value of 1.25 and a t-value of 10.42 (p< 0.001), represents the baseline level of help-seeking behavior when the effects of all independent variables are held at zero. This statistical foundation adds clarity to the overall predictive model. Furthermore, the model summary shows an R² value of 0.68, indicating that 68% of the variance in psychiatric help-seeking behavior is explained by the combined effects of cultural narratives, mental health stigma, and social norms. The adjusted R² of 0.67 accounts for the number of predictors and confirms the robustness and explanatory strength of the model. Additionally, the F-statistic of 85.42 (p< 0.001) confirms that the regression model as a whole is statistically significant and offers strong predictive validity.

In testing Hypothesis four, the findings provide strong empirical support for the alternative hypothesis (H_4_), confirming that the combined effects of cultural narratives, mental health stigma, and social norms significantly predict help-seeking behavior among individuals in Peshawar. These results reinforce the study’s conceptual framework, which positions mental health help-seeking as a multifactorial behavior shaped by cultural meaning systems, social pressures, and individual-level stigma perceptions. Ultimately, the findings underscore the necessity of multidimensional mental health interventions that not only challenge stigma but also reshape cultural narratives and promote positive social norms within the local context of Peshawar, Pakistan.

#### 3.3.2 Testing hypothesis H_4_: combined effects of cultural narratives, mental health stigma, and social norms on psychiatric help-seeking behavior

The results in **Table 6** derived from the SEM analysis offer an in-depth exploration of the direct and indirect influences of cultural narratives, mental health stigma, and social norms on psychiatric help-seeking behavior in the socio-cultural context of Peshawar, Pakistan. This analysis directly tests Hypothesis four (H_4_), which posits that the combined effects of these variables significantly predict help-seeking behavior. The model’s fit indices further reinforce the statistical soundness and theoretical coherence of the findings, confirming alignment with the study’s conceptual framework.

**Table 6:** Path analysis and SEM (multivariate).

Path coefficients				
Path	Standardized coefficient (β)	Standard error (SE)	Critical ratio (CR)	P-value
Cultural Narratives → Seeking Psychiatric Help	0.26	0.08	3.25	0.001
Mental Health Stigma → Seeking Psychiatric Help	-0.38	0.09	-4.22	0.000
Social Norms → Seeking Psychiatric Help	0.45	0.06	7.50	0.000
Cultural Narratives → Mental Health Stigma	-0.29	0.07	-4.14	0.000

Indirect effects				
Mediation path			Indirect effect (β)	P-value
Cultural Narratives → Mental Health Stigma → Seeking Psychiatric Help			0.11	0.003
Social Norms → Mental Health Stigma → Seeking Psychiatric Help			0.17	0.002

Model fit indices				
Fit index			Value	Threshold for good fit
Chi-Square (χ²)			78.65	p > 0.05
Degrees of Freedom (df)			45	N/A
χ²/df Ratio			1.75	< 3.0
Comparative Fit Index (CFI)			0.95	> 0.95
Tucker-Lewis Index (TLI)			0.93	> 0.90
RMSEA			0.043	< 0.06
SRMR			0.038	< 0.08

The direct path coefficients reveal significant and meaningful relationships between the independent variables and help-seeking behavior. Specifically, cultural narratives have a positive and statistically significant impact on the likelihood of seeking psychiatric help, as evidenced by a standardized coefficient (β) of 0.26, a critical ratio (CR) of 3.25, and a p-value of 0.001. This result suggests that individuals in Peshawar who are exposed to culturally supportive and health-affirming narratives are more inclined to access mental health services. In a setting where cultural traditions strongly influence individual behavior, this finding confirms that culturally embedded stories and values play a pivotal role in shaping attitudes toward psychiatric assistance.

Mental health stigma shows a substantial and statistically significant negative direct effect on help-seeking behavior, with a standardized coefficient (β) of -0.38, a CR of -4.22, and a p-value of 0.000. This confirms that stigma acts as a serious barrier to psychiatric help-seeking in Peshawar, where mental illness is often misunderstood and socially marginalized. This result is consistent with global and regional literature, and it substantiates the study’s theoretical position that stigma must be addressed to improve mental health service utilization.

Social norms emerge as a strong and significant positive predictor of help-seeking behavior, with a standardized coefficient (β) of 0.45, a CR of 7.50, and a p-value of 0.000. In the tightly knit communities of Peshawar, where societal expectations and communal values heavily influence personal decisions, this finding illustrates how supportive social norms can actively encourage individuals to pursue mental health treatment. This reinforces the importance of cultivating collective attitudes that endorse mental well-being and help-seeking as socially acceptable behaviors.

The analysis of indirect effects through the mediating role of mental health stigma further clarifies the dynamics between these variables. The indirect pathway from cultural narratives to help-seeking through stigma shows a significant standardized coefficient (β) of 0.11 (p = 0.003), indicating that positive cultural narratives reduce stigma, which in turn enhances the likelihood of seeking help. Likewise, the indirect effect from social norms to help-seeking behavior through stigma is also significant (β = 0.17, p = 0.002), emphasizing that supportive norms contribute to lowering stigma and indirectly promote access to psychiatric services. These mediation results highlight stigma as a central mechanism through which cultural and social factors influence help-seeking behaviors, reinforcing its critical role in the model.

The model fit indices collectively confirm that the SEM model is well-specified and demonstrates a strong fit to the observed data. The chi-square statistic (χ²) is 78.65 with a non-significant p-value (p > 0.05), indicating no major discrepancies between the observed and hypothesized covariance structures. The χ²/df ratio is 1.75, which is well below the standard threshold of 3.0, denoting a good model fit. The Comparative Fit Index (CFI) is 0.95, meeting the preferred criterion, and the Tucker-Lewis Index (TLI) is 0.93, surpassing the acceptable threshold of 0.90. The Root Mean Square Error of Approximation (RMSEA) is 0.043, and the Standardized Root Mean Square Residual (SRMR) is 0.038, both within the acceptable ranges of<0.06 and<0.08 respectively. These indicators collectively validate the model’s structure and affirm the reliability of the findings.

Based on the direct and indirect pathways and the overall model fit, the results provide strong empirical support for the hypothesis four (H_4_) that the combined effects of cultural narratives, mental health stigma, and social norms significantly predict help-seeking behavior. These findings validate the core assumptions of the study’s conceptual framework and demonstrate that in the context of Peshawar, psychiatric help-seeking behavior is deeply rooted in cultural understandings, societal norms, and the presence or absence of stigma. This underlines the need for culturally responsive mental health interventions that promote positive narratives, foster supportive social environments, and actively work to dismantle stigma.

## 4 Discussions

This study aimed to investigate how cultural narratives and social norms influence help-seeking behaviors for mental health issues in Peshawar, Khyber Pakhtunkhwa, with a specific focus on the mediating role of mental health stigma. The discussion reflects this aim by examining the socio-cultural, demographic, and psychological dimensions that shape perceptions and responses to mental illness within the local context. Mental health stigma—treated in this study both as a dependent variable (shaped by cultural narratives and social norms) and as a mediator influencing help-seeking behavior—plays a pivotal role in understanding the barriers to psychiatric care in conservative, collectivist societies.

The socio-economic and demographic characteristics of respondents significantly informed the complex interplay among cultural beliefs, stigma, and help-seeking decisions. These variables were not merely descriptive but interacted closely with the independent constructs of the study to shape behavioral outcomes.

Age emerged as a significant factor in shaping attitudes toward mental illness. Younger individuals, more exposed to education and global discourses, tend to adopt less stigmatizing views, showing greater mental health literacy (7, 31). In contrast, older respondents—more influenced by traditional Pashtunwali values—often interpret mental illness through spiritual or moral frameworks, reinforcing stigma (8, 24). This age-based divergence supports the study’s central model, where cultural narratives influence stigma, which in turn impacts help-seeking behavior.

Gender also shaped perceptions significantly. Women faced dual layers of stigma—both for experiencing mental health issues and for deviating from gendered expectations of emotional silence and resilience (1, 32). Conversely, men often avoided psychiatric help due to hegemonic masculinity norms associating emotional vulnerability with weakness, reinforcing internalized stigma (33, 34). This pattern illustrates how social norms directly influence both stigma and help-seeking behaviors, supporting the dual role of stigma within the study’s conceptual framework.

Education level was a strong predictor of mental health awareness and openness to formal intervention. Participants with higher education demonstrated reduced stigma and greater likelihood of seeking help, likely due to exposure to the biopsychosocial model of mental illness (21). This finding aligns with Ajzen (35) Theory of Planned Behavior, where enhanced knowledge reshapes subjective norms and perceived behavioral control.

Income level further influenced both perceptions and access to care. Individuals from higher-income backgrounds showed lower stigma and higher service utilization, likely due to improved healthcare access and reduced dependence on traditional or spiritual healing (8, 25). Low-income individuals, however, were more prone to internalized stigma and supernatural interpretations of mental illness, reinforcing harmful cultural narratives (5, 36).

Employment status contributed to the stigma dynamic by reflecting social utility. In Peshawar’s cultural context—where fulfilling one’s economic role is tightly linked with identity—unemployment intensified stigma and reduced help-seeking legitimacy (2, 23). These respondents faced stigmatization for not only being mentally unwell but also failing to meet expected societal roles, a dual burden corroborated by recent work on role-based stigma (21).

Bivariate regression analysis revealed that cultural narratives significantly predicted help-seeking behavior, explaining 42% of the variance. This confirms that culturally rooted belief systems, such as those embedded in Pashtunwali and Islamic ethics, strongly shape mental health perceptions. These narratives can either pathologize mental illness or, when positively framed, normalize it and encourage treatment (37, 38). In Peshawar’s collectivist context, shared stories of resilience, communal support, and spiritual understanding often mediate how individuals interpret mental illness and decide whether to seek help (39, 40).

The study’s logistic regression analysis demonstrated that mental health stigma is a significant barrier to help-seeking (β = -0.30, OR = 0.74), validating its mediating role within the model. While stigma was originally shaped by cultural narratives and social norms (making it a dependent variable), its impact on suppressing help-seeking behavior confirms its function as a mediating influence. This dual role aligns with the study’s theoretical foundation and builds on Corrigan et al (33) findings that fear of labeling and discrimination suppresses psychiatric service use, particularly in culturally conservative regions.

A notable contribution of this study is its emphasis on contextual, interpersonal sources of stigma. While much of the literature focuses on institutional or policy-related stigma, this research underscores how familial interactions, religious discourse, and social conformity reinforce stigma at the community level in Peshawar (31, 34). The findings support urgent calls for localized stigma reduction strategies rooted in cultural education and narrative reshaping.

Social norms were also found to be a strong positive predictor of help-seeking behavior (β = 0.42), consistent with Ajzen (35) Theory of Planned Behavior. In tight-knit, collectivist communities like Peshawar, decisions around health are often influenced by community expectations, religious authority, and family elders. This finding supports Gulliver et al (41) assertion that norm-driven behavior is especially salient in societies where communal interdependence governs decision-making.

Importantly, mediation analysis confirmed that social norms influence help-seeking not only directly but also indirectly by reducing stigma. This dual pathway demonstrates the transformative power of normative discourse in reshaping public attitudes. Glick et al. (42) similarly found that community-led interventions, which shift prevailing norms, can reduce stigma and promote mental healthcare uptake, especially in resource-limited settings (43).

### 4.1 Unique contributions and research gaps

This study makes several novel contributions to the mental health literature, particularly within under-researched South Asian and Muslim-majority contexts. It reveals how indigenous cultural structures like Pashtunwali, in combination with Islamic teachings and evolving global influences, shape psychological attitudes and behaviors.

### 4.2 Research gaps addressed:

- *Narrative Evolution in Transitional Societies*: The study examines how younger populations, influenced by education and digital exposure, increasingly adopt progressive attitudes, contrasting with older generations’ adherence to traditional moral interpretations (20, 24).
- *Stigma Reduction Through Cultural Levers*: Instead of clinical or institutional responses, the study identifies *culturally embedded levers*—like religious leaders, family elders, and community narratives—as powerful tools for reducing stigma and promoting help-seeking.
- *Localized Understanding of Norms*: The study highlights the specific mechanisms by which *social norms are enforced in a patriarchal, honor-based society*. It emphasizes how conformity to these norms not only shapes individual attitudes but defines legitimacy for engaging in psychiatric care (21, 29).

## 5 Conclusion

The analysis highlights the profound influence of cultural narratives, mental health stigma, and social norms on help-seeking behavior for psychiatric services in Peshawar, Khyber Pakhtunkhwa. Cultural narratives stand out as a pivotal factor, positively shaping individuals’ willingness to seek mental health support by fostering a climate of openness and reducing hesitation toward psychiatric care. This underscores the critical need for culturally sensitive mental health interventions that respect and integrate local traditions and belief systems, particularly in regions where cultural values strongly inform societal attitudes toward mental illness. Conversely, mental health stigma remains a major obstacle, significantly diminishing the likelihood that individuals will seek help. Stigma perpetuates fear, shame, and discrimination, deterring many from addressing their mental health needs. The findings reinforce the urgent requirement for targeted public awareness campaigns and educational initiatives aimed at dispelling myths and correcting misconceptions, especially in contexts where traditional beliefs exacerbate stigma. Furthermore, social norms emerge as a vital influence, with supportive community attitudes encouraging psychiatric help-seeking both directly and indirectly by mitigating stigma. These results reflect the unique cultural and social fabric of Peshawar and the wider Khyber Pakhtunkhwa region, where community expectations and social pressures play a central role in shaping individual health behaviors.

Collectively, these findings advocate for a holistic, culturally grounded approach to mental health care—one that addresses stigma, leverages positive cultural narratives, and engages social norms—to improve mental health service utilization in culturally complex and resource-constrained settings like Peshawar.

## 6 Policy implications

The findings of this study provide valuable insights into the critical role of cultural narratives, mental health stigma, and social norms in influencing help-seeking behavior for psychiatric care. Based on these results, the following policy implications are recommended:

### 6.1 Promoting mental health awareness campaigns

Policies should prioritize the design and implementation of culturally sensitive mental health awareness campaigns. These campaigns should aim to challenge harmful cultural narratives and promote positive perceptions of mental health services. By integrating culturally relevant storytelling and role models, such campaigns can enhance public understanding and acceptance of mental health care.

### 6.2 Reducing mental health stigma

Given the significant barrier posed by stigma, policymakers should focus on targeted stigma-reduction programs. Community-level interventions, such as peer-led initiatives, public endorsements by influential figures, and integration of mental health education into school curricula, can gradually change attitudes and normalize mental health care.

### 6.3 Strengthening social support systems

The role of social norms in facilitating help-seeking behavior underscores the need for policies that foster supportive community environments. Local governments and community organizations should invest in creating safe spaces for discussions around mental health and providing support groups to encourage help-seeking.

### 6.4 Capacity building for mental health services

Policies must ensure that mental health services are accessible, affordable, and adequately staffed with culturally competent professionals. Training mental health practitioners in understanding and addressing cultural and social factors is essential for effective service delivery.

### 6.5 Integration of mental health into primary healthcare

Incorporating mental health services into existing primary healthcare systems can reduce stigma and improve accessibility. Policymakers should work to establish mental health units within hospitals and clinics, particularly in underserved areas, to make psychiatric help more readily available.

### 6.6 Community-led interventions

Community-driven approaches should be supported to address cultural and social barriers to seeking help. Collaborative efforts with local leaders, religious scholars, and social influencers can encourage communities to reevaluate harmful norms and foster acceptance of mental health care.

### 6.7 Legislative measures

Enacting and enforcing policies that protect individuals against discrimination based on mental health conditions is crucial. Legal frameworks should also mandate the inclusion of mental health coverage in health insurance plans to alleviate financial barriers.

### 6.8 Public-private partnerships

Policymakers should engage private organizations and NGOs in mental health initiatives. Collaborative projects can provide innovative solutions and ensure the sustainability of mental health programs.

### 6.9 Research and data collection

Continuous research is necessary to monitor the effectiveness of implemented policies and programs. Policymakers should allocate resources to collect region-specific data on mental health trends and the socio-cultural factors influencing them.

### 6.10 Crisis intervention services

Establishing easily accessible crisis intervention services, such as 24/7 helplines and mobile mental health units, can address urgent mental health needs while reducing stigma and promoting help-seeking behaviors.

## 7 Strengths, limitations, and future directions

This study offers several important strengths that contribute valuable insights into the complex interplay between cultural narratives, stigma, social norms, and mental health help-seeking behavior in Peshawar, Khyber Pakhtunkhwa. By focusing on a culturally specific context, it provides nuanced understanding of how deeply embedded traditions and community expectations influence mental health attitudes and service utilization. The incorporation of multiple socio-cultural variables enriches the analysis and highlights pathways for culturally sensitive mental health interventions in under-resourced settings.

However, the study also has limitations that warrant consideration. First, the use of cross-sectional data restricts the ability to draw causal inferences between cultural narratives, stigma, social norms, and help-seeking behavior. Future research employing longitudinal designs could better capture the dynamic changes in attitudes and behaviors over time, thus clarifying causal pathways.

Second, the sample was drawn exclusively from a single socio-cultural setting, which may limit the generalizability of the findings to other regions or populations with different cultural or socio-economic characteristics. Expanding future research to include diverse cultural and geographic contexts would improve the broader applicability of the results.

Third, social desirability bias may have influenced participants’ responses, as individuals might have provided answers they deemed socially acceptable rather than fully candid views. To address this, future studies should incorporate qualitative methods, such as in-depth interviews or focus groups, to capture more authentic and diverse perspectives while mitigating response biases.

Lastly, while this study primarily focused on cultural narratives and stigma, it only briefly addressed structural barriers like financial constraints and limited access to mental health care. Future research should explore how these structural factors interact with socio-cultural influences to create a more comprehensive understanding of help-seeking behavior and inform holistic intervention strategies.

## Data Availability

The original contributions presented in the study are included in the article/supplementary material. Further inquiries can be directed to the corresponding author.

## References

[1] Taylor JM Gilligan C Sullivan AM. Between voice and silence: Women and girls, race and relationship. Cambridge, Massachusetts (MA), USA: Harvard University Press (1995).

[2] Donaldson NM. Black clinicians’ Perspectives on culturally relevant psychoeducation to increase mental health utilization. Pleasant Hill, California, USA: John F. Kennedy University (2022).

[3] Pasupuleti RV. Cultural factors, stigma, stress, and help-seeking attitudes among college students. Minneapolis, Minnesota, USA: Walden University (2013).

[4] Minaya C. The impact of structural stigma on OCD: symptom presentation, help-seeking, and quality of life fordham university. New York, New York, USA: Fordham University (2023).

[5] Mascayano F Armijo JE Yang LH. Addressing stigma relating to mental illness in low-and middle-income countries. Front Psychiatry. (2015) 6:38. doi: 10.3389/fpsyt.2015.00038, PMID: 25814959 PMC4355984

[6] Patel V. Mental health in low-and middle-income countries. Br Med Bull. (2007) 81:81–96. doi: 10.1093/bmb/ldm010, PMID: 17470476

[7] Begum R Choudhry FR Khan TM Bakrin FS Al-Worafi YM Munawar K. Mental health literacy in Pakistan: A narrative review. Ment Health Rev J. (2020) 25:63–74. doi: 10.1108/MHRJ-08-2019-0026

[8] Saeed K Gater R Hussain A Mubbashar M. The prevalence, classification and treatment of mental disorders among attenders of native faith healers in rural Pakistan. Soc Psychiatry Psychiatr Epidemiol. (2000) 35:480–5. doi: 10.1007/s001270050267, PMID: 11127723

[9] Rashid A Mudassar U Tariq I Zaheer A Iftikhar M Mazhar N. Correlation of depression, anxiety and stress with quality of life in COVID-19 pandemic. Esculapio. (2021) 17:195–199.

[10] Waqas A Zubair M Ghulam H Ullah MW Tariq MZ. Public stigma associated with mental illnesses in Pakistani university students: a cross-sectional survey. PeerJ. (2014) 2:e698., PMID: 25548734 10.7717/peerj.698 PMC4273937

[11] Al-Farsi OA Al-Farsi YM Al-Sharbati MM Al-Adawi S. Stress, anxiety, and depression among parents of children with autism spectrum disorder in Oman: a case–control study. Neuropsychiatr Dis Treat. (2016) 12:1943–1951. doi: 10.2147/NDT.S107103, PMID: 27536117 PMC4977076

[12] Koenig HG. Religion, spirituality, and health: a review and update. Adv Mind‑Body Med. (2015) 29:19–26. 26026153

[13] Youssef J Deane FP. Factors influencing mental-health help-seeking in Arabic-speaking communities in Sydney, Australia. Ment Health Relig Cult. (2006) 9:43–66. doi: 10.1080/13674670512331335686

[14] Khan G Khan DN Shafqat I Shah H Khawaja I Aftab A. Prevalence of anxiety and depression among celiac disease patients. Int J Health Sci. (2023) 7:1668–1675. doi: 10.53730/ijhs.v7nS1.14395

[15] Khan MN Javed MA. Teaching & training in psychiatry: Pakistani perspectives. In: Sathyanarayana Rao TS, editor. Psychiatry in India: Training & Training Centres (Revised & Updated Edition). Indian Journal of Psychiatry. 2nd ed. (2015).

[16] Hussain K Ahmed T. Social media usage and its relationship with depression among nursing students of a private university: social media usage in relationship with depression. Pak Biomed J. (2024) 13–17.

[17] Ali RK. Exploring bullying aganist academics in Pakistan: A corrosive workplace issue The University of Waikato. Hamilton, New Zealand: The University of Waikato (2024).

[18] Zulfiqar N Hafeez S Habib A. Quality of life among caregivers of psychiatric patients: educational qualification and gender differences. Glob Educ Stud Rev. (2020) 3:426–434.

[19] Rosli AM Hashi AA Razali ZA. Jinn and mental illness among Muslims—a commentary. IIUM Med J Malays. (2020) 19.

[20] Ahmad S Bano A. Professionals unprepared: A critical appraisal of social work practice at the Drugs Abuse Rehabilitation Centres in Khyber Pakhtunkhwa, Pakistan. J Humanities Soc Manage Sci (JHSMS). (2021) 2:108–20. doi: 10.47264/idea.jhsms/2.1.10

[21] Choudhry FR Khan N Munawar K. Barriers and facilitators to mental health care: A systematic review in Pakistan. Int J Ment Health. (2023) 52:124–62. doi: 10.1080/00207411.2021.1941563

[22] Maloušková K Fafejta M. The social marginalization of people living with a mentally ill label–family, friends, and work. Qual Sociology Rev. (2021) 17:76–89. doi: 10.18778/1733-8077.17.3.04

[23] Yang LH Thornicroft G Alvarado R Vega E Link BG. Recent advances in cross-cultural measurement in psychiatric epidemiology: utilizing ‘what matters most’to identify culture-specific aspects of stigma. Int J Epidemiol. (2014) 43:494–510. doi: 10.1093/ije/dyu039, PMID: 24639447 PMC13387039

[24] Gregg GS. The Middle East: a cultural psychology. New York, New York, USA: Oxford University Press (2005).

[25] Rathod S Pinninti N Irfan M Gorczynski P Rathod P Gega L. Mental health service provision in low-and middle-income countries. Health Serv Insights. (2017) 10:1178632917694350. doi: 10.1177/1178632917694350, PMID: 28469456 PMC5398308

[26] Onwuameze NC. Educational opportunity and inequality in Nigeria: assessing social background, gender, and regional effects. Iowa City, Iowa, USA: The University of Iowa (2013).

[27] Lim A Hoek HW Ghane S Deen M Blom JD. The attribution of mental health problems to jinn: an explorative study in a transcultural psychiatric outpatient clinic. Front Psychiatry. (2018) 9:89., PMID: 29643820 10.3389/fpsyt.2018.00089 PMC5882841

[28] Perkins HW Berkowitz AD. Perceiving the community norms of alcohol use among students: some research implications for campus alcohol education programming. Int J Addict. (1986) 21:961–976., PMID: 3793315 10.3109/10826088609077249

[29] Huesmann LR Moise-Titus J Podolski CL Eron LD. Longitudinal relations between children's exposure to TV violence and their aggressive and violent behavior in young adulthood: 1977–1992. Dev Psychol. (2003) 39:201., PMID: 12661882 10.1037//0012-1649.39.2.201

[30] Day A Chung D O’Leary P Carson E. Programs for men who perpetrate domestic violence: an examination of the issues underlying the effectiveness of intervention programs. J Fam Violence. (2009) 24:203–212.

[31] Wei Y McGrath PJ Hayden J Kutcher S. Mental health literacy measures evaluating knowledge, attitudes and help-seeking: a scoping review. BMC Psychiatry. (2015) 15:1–20. doi: 10.1186/s12888-015-0681-9, PMID: 26576680 PMC4650294

[32] Shankar A. Examining the role that social abjection and stigma play in prohibiting female re-acceptance into minority cultures post wartime-rape macquarie university. Sydney, Australia: Macquarie University (2023).

[33] Corrigan PW Druss BG Perlick DA. The impact of mental illness stigma on seeking and participating in mental health care. psychol Sci Public Interest. (2014) 15:37–70. doi: 10.1177/1529100614531398, PMID: 26171956

[34] Vogel DL Wade NG. The Cambridge handbook of stigma and mental health. Cambridge, United Kingdom: Cambridge University Press (2022).

[35] Ajzen I. The Theory of planned behavior. In: Organizational behavior and human decision processes. San Diego, California, USA: Elsevier (1991).

[36] Bhugra D. Mental health for nations. Int Rev Psychiatry. (2016) 28:342–74. doi: 10.1080/09540261.2016.1211095, PMID: 27686156

[37] Kleinman A. Patients and healers in the context of culture: An exploration of the borderland between anthropology, medicine, and psychiatry Vol. 3. Berkeley, California, USA: Univ of California Press (1980).

[38] Sheikh S Furnham A. A cross-cultural study of mental health beliefs and attitudes towards seeking professional help. Soc Psychiatry Psychiatr Epidemiol. (2000) 35:326–34. doi: 10.1007/s001270050246, PMID: 11016528

[39] Sum MY Wong CTW Chu ST Li A Lee AHT Chen EYH. Systematic review and meta-analysis of internalised stigma and stigma resistance in patients with psychosis: The impact of individualism-collectivism culture and other individual factors. Int J Soc Psychiatry. (2024) 70:639–52. doi: 10.1177/00207640231216924, PMID: 38279534

[40] Sullivan PL. Culture, divorce, and family mediation in Hong Kong. Family Court Rev. (2005) 43:109–23. doi: 10.1111/j.1744-1617.2005.00011.x

[41] Gulliver A Griffiths KM Christensen H. Barriers and facilitators to mental health help-seeking for young elite athletes: a qualitative study. BMC Psychiatry. (2012) 12:1–14. doi: 10.1186/1471-244X-12-157, PMID: 23009161 PMC3514142

[42] Glick AR Jones C Martignetti L Blanchette L Tova T Henderson A. An integrated empirical and computational study to decipher help-seeking behaviors and vocal stigma. Commun Med. (2024) 4:228. doi: 10.1038/s43856-024-00651-3, PMID: 39521864 PMC11550451

[43] Daraz U Khan Y Alsawalqa RO Alrawashdeh MN Alnajdawi AM. Impact of climate change on women mental health in rural hinterland of Pakistan. Front Psychiatry. (2024) 15:1450943. doi: 10.3389/fpsyt.2024.1450943, PMID: 39735428 PMC11674845

